# A single-arm study design with non-inferiority and superiority time-to-event endpoints: a tool for proof-of-concept and de-intensification strategies in breast cancer

**DOI:** 10.3389/fonc.2023.1048242

**Published:** 2023-07-11

**Authors:** Miguel Sampayo-Cordero, Bernat Miguel-Huguet, Andrea Malfettone, Elena López-Miranda, María Gion, Elena Abad, Daniel Alcalá-López, Jhudit Pérez-Escuredo, José Manuel Pérez-García, Antonio Llombart-Cussac, Javier Cortés

**Affiliations:** ^1^ Medica Scientia Innovation Research (MEDSIR), Barcelona, Spain; ^2^ Gerència Territorial Metropolitana Sud, Institut Català De La Salud, Hospital Universitari De Bellvitge, Barcelona, Spain; ^3^ Hospital Universitario Ramón y Cajal, Madrid, Spain; ^4^ International Breast Cancer Center (IBCC), Quiron Group, Barcelona, Spain; ^5^ Hospital Arnau de Vilanova, FISABIO, Universidad Católica de Valencia, Valencia, Spain; ^6^ Vall d´Hebron Institute of Oncology (VHIO), Barcelona, Spain

**Keywords:** time-to-event, non-inferiority, single-arm, phase II, clinical trial, superiority

## Abstract

De-escalation trials in oncology evaluate therapies that aim to improve the quality of life of patients with low-risk cancer by avoiding overtreatment. Non-inferiority randomized trials are commonly used to investigate de-intensified regimens with similar efficacy to that of standard regimens but with fewer adverse effects (ESMO evidence tier A). In cases where it is not feasible to recruit the number of patients needed for a randomized trial, single-arm prospective studies with a hypothesis of non-inferiority can be conducted as an alternative. Single-arm studies are also commonly used to evaluate novel treatment strategies (ESMO evidence tier B). A single-arm design that includes both non-inferiority and superiority primary objectives will enable the ranking of clinical activity and other parameters such as safety, pharmacokinetics, and pharmacodynamics data. Here, we describe the statistical principles and procedures to support such a strategy. The non-inferiority margin is calculated using the fixed margin method. Sample size and statistical analyses are based on the maximum likelihood method for exponential distributions. We present example analyses in metastatic and adjuvant settings to illustrate the usefulness of our methodology. We also explain its implementation with nonparametric methods. Single-arm designs with non-inferiority and superiority analyses are optimal for proof-of-concept and de-escalation studies in oncology.

## 1 Introduction

Molecular diagnostics and biomarkers have enabled many cancers to be divided into clinical and biological subtypes, some of which have a low risk of relapse or death (1–4). In patients with low-risk breast cancer, de-escalation trials are increasingly being conducted to evaluate therapies that aim to improve quality of life by avoiding overtreatment (1, 5, 6). These trials use non-inferiority designs to investigate de-intensified regimens with efficacy similar to that of standard treatments but with fewer toxic effects (5, 7–9). Although randomized trials provide the strongest evidence (ESMO evidence tier A) for the efficacy of de-escalation strategies (10–13), randomized designs are not always the most efficient option and cannot be used to answer all research questions (14–18). Furthermore, in certain cancer types and phases of clinical development, it is not feasible to recruit the number of patients needed for a randomized clinical trial. In such cases, de-escalation strategies can be investigated using single-arm or non-comparative trials (ESMO evidence tier B) (6, 13, 19–22). Single-arm trials can also be used to evaluate novel therapies, agents with a high expectation of tumor response, rare cancers, salvage therapies, and therapies for late-stage disease, especially when no standard-of-care exists and a robust historical database is available (15, 18, 23). The inclusion of both non-inferiority and superiority primary objectives in single-arm study designs enables informed decisions that rank the magnitude of clinical activity along with other parameters such as safety, pharmacokinetics, and pharmacodynamics data (24–26).

Some treatments have been successful in phase III trials even after producing negative results in phase II single-arm trials. In these situations, a new treatment was deemed non-inferior to standard-of-care therapy when considered in the context of relevant parameters such as safety, duration of clinical benefit, or targeting of a new biological pathway (27, 28). However, the likelihood of a type I error (α) increases when a *post-hoc* non-inferiority analysis is performed after an unsuccessful proof-of-concept trial (25, 29). The probability of such an error can be reduced by including the non-inferiority analysis in the experimental design *a priori* (24–26). It is easy to include non-inferiority and superiority analyses in single-arm one-stage or two-stage studies with response rate as the primary endpoint (24); however, the most reliable and preferred endpoint in cancer studies is overall survival.

It is common to plan proof-of-concept and confirmatory studies in oncology using time-to-event endpoints (19, 30, 31). Most approvals for breast cancer drugs in adjuvant and advanced settings are supported by improvements in overall survival, disease-free survival (DFS), and progression-free survival (PFS) (32). Although there are a few single-arm trials that used a historic control arm to set a non-inferiority threshold for a time-to-event outcome (5, 20, 33), these trials did not include and additional superiority analysis for the primary objective. Here, we propose a single-arm, time-to-event study design that includes both superiority and non-inferiority analyses.

## 2 Materials and methods

### 2.1 Non-inferiority margin

Single-arm studies with a time-to-event primary endpoint usually include a superiority analysis that aims to show that the probability of survival (e.g., median PFS [mPFS]) with a certain treatment is greater than the probability of survival estimated for an active control arm (mPFS0) in a previous trial (34). Conversely, the risk of progression or death with the treatment, represented by a hazard rate (λ) equal to the Napierian logarithm of 2 (LN[2]) divided by mPFS, is expected to be lower than the risk of progression or death in the active control arm (λ0) (34). In contrast to such superiority analyses, a non-inferiority analysis aims to show that the effect of a test drug in terms of survival is not inferior to that of the historical comparator by more than a specified amount called the non-inferiority margin (NIM) (29). The NIM calculation is based on the difference in observed effects between the historical comparator and placebo in previous studies, which is represented by a hazard ratio (HR) that is greater than 1 and equal to either the mPFS0 divided by the mPFS in the placebo arm (mPFS_placebo_) or the λ in the placebo arm (λ_placebo_) divided by the λ0 (7, 24, 29). For example, if the HR is 2.4 with a 95% confidence interval (CI) of 1.44–3.56, the fixed margin method is applied to select the 95% CI lower bound (1.44) and adjust it to retain at least 50% of the historical effect of the active control versus the placebo: 1.44^(1–0.5)^ = 1.2. Accordingly, the calculated NIM describes a ratio reflecting the largest loss of the effect previously observed in the active control arm that would be clinically acceptable (29).

### 2.2 Non-inferiority and superiority analyses in a single-arm design

The null hypothesis (H0) for superiority and non-inferiority analyses in a one-sided test can be defined in terms of survival (mPFS) or hazard (λ) parameters as follows:


(1) $${H0}_{\text{superiority}}:mPFS\leq mPFS0;\lambda \geq \lambda 0$$

(2) $${H0}_{\text{non-inferiority}}\text{:mPFS≤}\left(mPFS0/NIM\right)\text{;λ}\geq \text{(λ0 x NIM)}$$

Additionally, the magnitude of the difference between the treatment arm and the historical control (i.e., the effect size) can be defined in terms of mPFS or λ for superiority and non-inferiority analysis as follows:


(3) Superiority: mPFS – mPFS0; λ – λ0

(4) Non-inferiority: mPFS – (mPFS0 / NIM); λ – (λ0xNIM)

The cutoff for H0_non-inferiority_ will be always lower than the cutoff for H0_superiority_ (i.e., mPFS0_non-inferiority_ < mPFS0_superiority_), and the converse will be true when H0 is defined in terms of hazard rates (i.e., λ0_non-inferiority_ > λ0_superiority_). At the time of final analysis in a single-arm trial, the number of patients recruited (n), the events observed, the mPFS, and the λ will be equal for the non-inferiority and superiority analyses. Therefore, the difference in effect size between the superiority and non-inferiority analyses is totally dependent on the magnitude of the preplanned H0. In a one-sided test that will accept H0 if the study mPFS is less than the mPFS of the historical control, H0 is rejected only if mPFS is greater than mPFS0 (or λ is less than λ0). In all these scenarios, the absolute value of the effect size in the non-inferiority analysis will be greater than the absolute value of the effect size in the superiority analysis:


(5) |mPFS – mPFS0|< |mPFS – (mPFS0 / NIM)|

The same is true in terms of hazard rates:


(6) If λ < λ0, then λ0 < (λ0 x NIM) and |λ – λ0|< |λ – (λ0 x NIM)|

As the number of events is equal in the superiority and non-inferiority analyses, it follows that the probability of detecting an effect (i.e., the power of the test) will always be greater in the non-inferiority analysis than in the superiority analysis. Therefore, the type II error level (β) planned for the superiority analysis is retained in the inferiority analysis (24, 35).

As stated in the United States Food and Drug Administration’s multiple endpoint guidelines, despite evaluating multiple hypotheses, “after demonstrating non-inferiority on the endpoint, it is possible to then test for superiority at an unadjusted alpha” Thus, in a superiority analysis with a time-to-event primary endpoint, analysis of a non-inferiority hypothesis does not inflate the type I error rate when the non-inferiority analysis and NIM are properly pre-specified (29, 36).

The design proposed here can be used to assess both superiority and non-inferiority criteria with the same sample size, type I error rate (α), and β that would be used in a superiority-only strategy. This applies to both parametric (exponential or Weibull distribution estimator) and nonparametric (Kaplan–Meier or life table estimator) approaches (34, 37).

## 3 Results

### 3.1 Sample size calculation in a metastatic setting

The following section provides a numerical example of the proposed design for a typical phase II single-arm (proof-of-concept) trial that includes both non-inferiority and superiority analyses in a metastatic setting. Suppose that mPFS for a standard therapy is 12 months. This corresponds to a λ0 (LN[2]/mPFS[12]) of 0.058. We would design a study to detect mPFS improvement to at least 18 months (λ1 = LN[2]/18 = 0.039), producing an HR of 0.67 (HR = 12/18). We plan a 12-month accrual period (ap) and a 24-month follow-up period (fp). We design the study to attain 90% power (1 – β) using the maximum likelihood method for exponential distributions at a nominal one-sided α level of 10%. The maximum accepted α level in our example is higher than what is usually used in confirmatory trials (i.e., one-sided α of 2.5% or two-sided α of 5%). This is appropriate because of the exploratory nature of our trial. We also assume a 10% dropout rate. The required number of patients and events is calculated as follows, where Z is the standard normal cumulative distribution function for a one-sided test (34):


(7) $$Events=\frac{{\left(Z_{1-\text{α}=0.1}+\;Z_{1-\text{β}=0.1}\right)}^{2}}{{\left(LN\left(HR=0.667\right)\right)}^{2}}=\frac{\;{\left(1.282\;+\;1.282\right)}^{2}}{{\left(-0.405\right)}^{2}}=39.96\;\approx \;40$$

$Probability\;ofevent=1-\left(\frac{e^{\left(-\text{λ}1\times \text{fp}\right)}}{\text{λ}1\times \text{ap}}\times \left(1-e^{\left(-\text{λ}1\times \text{ap}\right)}\right)\right)=$


(8) $$1-\left(\frac{e^{\left(-0.924\right)}}{1.39}\times \left(1-e^{\left(-0.462\right)}\right)\right)=0.682$$

(9) $$Number\;of\;Patients=\frac{Events\;}{Probability\;of\;event}=\;\frac{40\;}{0.682}=58.6$$

(10) Dropoutcorrection=58.6/(1–dropout rate [0.1])=65.1≈66 patients

The Excel functions to resolve this are “INV.NORM.ESTAND(1–(α=0.1))” and “INV.NORM.ESTAND(1–(β=0.1))”; e^X^ represents the natural exponential function (“exp(x)” in Excel).

### 3.2 Final analyses in a metastatic setting

Continuing with this example, it is supposed that by the end of the study 66 patients have been accrued, 54 PFS events have occurred, and the final mPFS is 12 months, with a hazard rate (λobs) of 0.058. Based on an NIM of 1.2, H0_non-inferiority_ is an mPFS (mPFS0/NIM = 12/1.2) of 10 months, which is equivalent to a non-inferiority hazard rate (λ_NI_ = LN[2]/10) of 0.069. Final statistical analyses for the superiority and non-inferiority objectives are performed using the maximum likelihood method for exponential distributions as follows (34):

*Non-inferiority:*

$\text{p value }=\;\left(1-\Phi \;\left(\sqrt{events}\times (LN\left(\text{λobs}\right)-\left(LN\left({\text{λ}}_{NI}\right)\right)\right)\right)\;=$

$\left(1-\Phi \;\left(\sqrt{54}\times (LN\left(0.058\right)-\left(LN\left(0.069\right)\right)\right)\right)=$


(11) 0.09 (p<0.1)

*Superiority:*

$\text{p value }=\;\left(1-\Phi \;\left(\sqrt{events}\times (LN\left(\text{λobs}\right)-\left(LN\left(\text{λ}0\right)\right)\right)\right)\;=$

$\left(1-\Phi \;\left(\sqrt{54}\times (LN\left(0.058\right)-\left(LN\left(0.058\right)\right)\right)\right)=$


(12) 0.5 (p>0.1)

The expression “ 1−Φ () “ is the standard normal cumulative distribution, which is used to back-transform Z-scores into p values.

### 3.3 Analysis in an adjuvant setting

Usually, the primary objective of clinical trials in adjuvant settings is DFS. The DFS rate is usually higher than 50%, so the median survival is not estimable. However, the previous analyses can be conducted in an adjuvant setting if the DFS rates are transformed into hazard rates and HRs. In a study investigating an adjuvant therapy in early-stage breast cancer by Cardoso et al. (2016), the DFS rate without distant metastasis for a standard therapy was 95% at 5 years (60 months). This corresponds to a λ0 of 0.0009 (5).


(13) λ0 = -LN(0.95)/(60 months)=0.0009

The pre-specified NIM corresponds to a 3% difference in DFS without distant metastases at 5 years (i.e., from 95% to 92%). This corresponds to a λ0_NI_ of 0.0014 (5).


(14) $${\lambda 0}_{\text{NI}}\text{= -LN}\left(0.92\right)/\left(60 months\right)=0.0014$$

The study was designed to attain 80% power at a nominal two-sided α level of 5%. Based on equation (7), the required number of events for this single-arm design is 34. By contrast, 135 events would be needed in a randomized study. The criteria for the primary analysis were met with 748 patients recruited from the primary test population; however, we would need about 3000 patients if we used a comparative design.

## 4 Discussion

De-intensification strategies are often developed in response to new diagnostics that can select patients who do not need aggressive therapy. Our proof-of-concept example shows how a novel de-intensified treatment can be shown to be non-inferior to the standard of care in selected patients. In such a case, if safety data show that the novel treatment is better tolerated than the standard-of-care, further confirmatory studies should be developed to evaluate the novel treatment. Our method can also be used to explore the efficacy of new drugs. For example, if a new drug achieves the non-inferiority objective, as in our proof-of-concept example, and also shows good tolerability, an appropriate pharmacokinetic profile, and/or a novel molecular target, then it might be expected to show further promising results when combined with standard treatment in a phase II/III randomized trial. Similarly, if a new drug achieves the superiority objective in our study design, this would suggest that it may be effective as a monotherapy (24).

Our approach enables the design of non-inferiority breast cancer studies in settings where it is not feasible to recruit enough patients for a comparative analysis (5, 6, 13). Although the sample size needed for a non-inferiority study is usually expected to be greater than the sample size needed for a superiority study, this is a misunderstanding. Actually, when a non-inferiority study uses the same assumptions as a superiority study, the non-inferiority study will always need fewer patients than the superiority study. The reason that non-inferiority studies are thought to need more patients than superiority studies is because non-inferiority studies assume that two treatments are equally effective, whereas superiority studies never make this assumption (38). In addition to the use of a single-arm primary analysis, there are some other ways to improve the quality of data in de-intensification studies. For example, a randomized non-comparative design can be used, in which the randomized control arm has far fewer patients than would be needed for a powered comparative analysis (5, 6). Other approaches might be to use an external control based on previous clinical trials, detailed cancer registries, real-world evidence, and synthetic control arms (39).

A single-arm trial designed to analyze both non-inferiority and superiority objectives enables ranking of early efficacy and other parameters such as safety, pharmacokinetics, and pharmacodynamics data (26), making such an approach more informative than a trial designed to analyze only superiority or non-inferiority (24–26). As non-inferiority analysis is allowed, it could be conjectured that this design makes easier that ineffective therapies were assessed as promising. This assumes that evaluation of the treatment is based exclusively on the non-inferiority result, without considering other objectives. Risk-benefit assessments weighing all endpoints are usually performed when establishing development plans for new drugs. Accordingly, our method supports a comprehensive approach to drug development by enabling the totality of the evidence to be considered in favor of a therapy (40, 41).

Non-inferiority analysis can be implemented in time-to-event studies using the log-rank test methodology (42). In addition, our proposed design can be easily implemented with nonparametric methods such as Kaplan–Meier analysis, which usually estimates median survival or survival rates based on CIs. For instance, we would achieve a positive non-inferiority result with a 90% CI of 3.6–5.6, because the lower bound of the CI would be greater than 3.3, the H0 for the non-inferiority test. Conversely, the superiority objective would not be achieved, because the lower bound of the 90% CI would be lower than 4, the H0 for the superiority test. The sample size in time-to-event designs based on nonparametric tests can be easily calculated with various methods and online calculators (43–45). The same strategy can be used to prespecify thresholds for null hypotheses under a Bayesian framework (31).

Our method includes the inherent drawbacks of studies that rely on historical controls and non-inferiority analyses. These limitations are common and well-known in non-inferiority comparative study designs because the NIM must be based on historical evidence (46). In randomized controlled designs of non-inferiority, it is necessary to demonstrate assay sensitivity to declare a therapy non-inferior in a single-arm trial (12). Additionally, selection of inappropriate patients, premature discontinuations, and poor compliance all favor conclusions of a lack of difference between experimental and control arms in randomized trials. This can lead to erroneous declarations of non-inferiority of the experimental treatment. This bias is reduced when comparisons are based on a theoretical rate of efficacy deduced from historical controls (12, 46). Accordingly, single-arm designs with non-inferiority analyses that are properly preplanned and conducted are not more challenging than the usual randomized or single-arm designs (5, 24).

Various strategies to evaluate multiple endpoints of efficacy and safety in proof-of-concept trials have been proposed in Bayesian and frequentist paradigms (e.g., EFFTox, Gumbel model, continual reassessment method, and single-stage and two-stage time-to-event designs). However, these designs do not rank non-inferiority and superiority hypotheses to grade the magnitude of clinical activity in the early clinical stages (19, 47–49).

Altogether, analyses of non-inferiority and superiority in single-arm trials are easily implemented in typical time-to-event designs for adjuvant and metastatic settings. This approach is useful for weighing additional factors such as safety, cost, and biomarkers while also assessing efficacy, making it optimal for proof-of-concept and de-intensification investigations in oncology.

## Data availability statement

The raw data supporting the conclusions of this article will be made available by the authors, without undue reservation.

## Author contributions

MS-C, AL-C, and JC conceived the study. MS-C, BM-H, DA-L, and EA developed the statistical methods. EL-M, MG, AM, JP-G, JP-E, AL-C, and JC reviewed the methods. MS-C, BM-H, DA-L, and EA conducted the statistical analysis. EL-M, MG, JP-G, AL-C, and JC reviewed the statistical results. MS-C, AM, and JP-G drafted the manuscript. All authors contributed to the article and approved the submitted version.
